# Small and big Hodgkin-Reed-Sternberg cells of Hodgkin lymphoma cell lines L-428 and L-1236 lack consistent differences in gene expression profiles and are capable to reconstitute each other

**DOI:** 10.1371/journal.pone.0177378

**Published:** 2017-05-15

**Authors:** Benjamin Rengstl, Sooji Kim, Claudia Döring, Christian Weiser, Julia Bein, Katrin Bankov, Marco Herling, Sebastian Newrzela, Martin-Leo Hansmann, Sylvia Hartmann

**Affiliations:** 1 Dr. Senckenberg Institute of Pathology, Goethe University, Frankfurt am Main, Germany; 2 Laboratory of Lymphocyte Signaling and Oncoproteome, Department of Internal Medicine I, University of Cologne, Cologne, Germany; 3 Center for Integrated Oncology (CIO) Köln-Bonn, CECAD, and CMMC, University of Cologne, Cologne, Germany; European Institute of Oncology, ITALY

## Abstract

The hallmark of classical Hodgkin lymphoma (cHL) is the presence of giant, mostly multinucleated Hodgkin-Reed-Sternberg (HRS) cells. Whereas it has recently been shown that giant HRS cells evolve from small Hodgkin cells by incomplete cytokinesis and re-fusion of tethered sister cells, it remains unsolved why this phenomenon particularly takes place in this lymphoma and what the differences between these cell types of variable sizes are. The aim of the present study was to characterize microdissected small and giant HRS cells by gene expression profiling and to assess differences of clonal growth behavior as well as susceptibility toward cytotoxic intervention between these different cell types to provide more insight into their distinct cellular potential. Applying stringent filter criteria, only two differentially expressed genes between small and giant HRS cells, *SHFM1* and *LDHB*, were identified. With looser filter criteria, 13 genes were identified to be differentially overexpressed in small compared to giant HRS cells. These were mainly related to energy metabolism and protein synthesis, further suggesting that small Hodgkin cells resemble the proliferative compartment of cHL. *SHFM1*, which is known to be involved in the generation of giant cells, was downregulated in giant RS cells at the RNA level. However, reduced mRNA levels of *SHFM1*, *LDHB* and *HSPA8* did not translate into decreased protein levels in giant HRS cells. In cell culture experiments it was observed that the fraction of small and big HRS cells was adjusted to the basic level several days after enrichment of these populations via cell sorting, indicating that small and big HRS cells can reconstitute the full spectrum of cells usually observed in the culture. However, assessment of clonal growth of HRS cells indicated a significantly reduced potential of big HRS cells to form single cell colonies. Taken together, our findings pinpoint to strong similarities but also some differences between small and big HRS cells.

## Introduction

The pathogenesis of classical Hodgkin lymphoma (cHL) has been unsolved for many years. Already around 1900, Dorothy Reed and Carl Sternberg were fascinated by the morphological appearance of the tumor cells, particularly by the usually giant bi- or multinucleated so called Reed-Sternberg (RS) cells [1, 2]. In 1994, it could first be demonstrated that these enigmatic Hodgkin and Reed-Sternberg (HRS) cells constitute a clonal B-cell population [3]. Although it was previously speculated that RS cells develop after fusion of cells [4], as known from histiocytic giant cells, single cell analyses revealed that these giant multinucleated cells never present more than two rearranged immunoglobulin genes [5], indicating that RS cells have probably developed from endomitosis as observed in the cHL cell line HDML-2 [6]. Recent studies discovered that giant multinucleated RS cells evolve from small mononucleated Hodgkin cells by incomplete cytokines and re-fusion of tethered sister cells [7]. However, there is also a subset of giant cells containing only one enormous nucleus and not resulting from a re-fusion [7]. In primary cHL samples and the cHL cell lines L-428, KM-H2, and HDLM-2 Hoechst dye-negative side populations—considered as tumor stem cells—could be identified [8, 9]. In culture experiments, these side populations were shown to be able to reconstitute the HRS clone, whereas giant binucleated RS cells failed to proliferate [8, 10]. However, these side populations only represent a small subset of the abundant small HRS cells observed in cell culture. Interestingly, particularly the cHL cell lines L-428 and L-1236 show mono- and multinucleated tumor cells of very variable sizes, including giant tumor cells with sometimes sizes above 100 μm in diameter. Consequently, the aim of the present study was to determine the differences in gene expression profiles, growth kinetics, clonal growth potential, and vulnerability towards treatment of small and giant HRS cells and to gain deeper insight into this particular phenomenon of populations of different cell sizes in cHL cell lines.

## Materials and methods

### Cell culture and laser microdissection

The cHL cell lines L-428 and L-1236 were obtained from the German Collection of Microorganisms and cell cultures (DSMZ, Braunschweig, Germany) and cultured in RPMI with 10% fetal calf serum. In first experiments, isolation of giant HRS cells in high purity by fluorescence associated cell sorting proved to be difficult, since these cells are very fragile to the sorting procedure. Therefore, laser microdissection was applied as method of choice. Two days after passage, the cells were washed and resuspended in 300 μl phosphate buffered saline substituted with 0.6 μl RNAse inhibitor. Smears of the cell lines were made on membrane slides for laser microdissection and air dried for 15 min. Microdissection was performed on a Microdissection Axiovert 200M microscope (PALM, Bernried, Germany). Cells to be considered as small HRS cells showed a maximum cell area of 350 μm^2^ in the PALM microscope, whereas big HRS cells had an area of more than 600 μm^2^. In order to obtain comparable amounts of RNA, the total microdissected areas of small and big HRS cells were adjusted to approximately 700 big and 1500 small HRS cells. Cells were catapulted in PALM adhesive caps and lysed with 2 ml NUGEN Direct Lysis Buffer (NUGEN, Bemmel, The Netherlands).

### Gene expression analysis

After lysis of the microdissected cells, RNA was amplified with the WT-Ovation-One-direct-Kit (NUGEN) and hybridized onto Affymentrix Gene Arrays 1.0 ST (Affymetrix, Santa Clara, CA, USA). Gene expression analysis was performed and analyzed as previously described [11, 12]. Gene expression data are available through the GEO database (GEO accession number GSE86477).

### Immunohistochemistry, fluorescence microscopy, and primary cases

Immunohistochemistry for SHFM1, LDHB and HSPA8 was performed using the FLEX-Envision Kit (DAKO, Glostrup, Denmark) as described previously [13]. Primary antibodies for SHFM1 (LS-B899, Lifespan Biosciences, Seattle WA, USA, dilution 1:50), LDHB (LS-B4366, Lifespan, dilution 1:100) and HSPA8 (1B5, Enzo Life Sciences, Lörrach, Germany, dilution 1:100) were applied over night at 4°C after heat induced epitope retrieval in citrate buffer (pH6) for 10 min. Primary cHL cases (5 mixed cellularity, 5 nodular sclerosing subtype) were selected from the archives of the Dr. Senckenberg Institute of Pathology, Frankfurt, Germany. The study was approved by the local ethics committee of the Frankfurt University Hospital.

For immunofluorescence, the cHL cell lines L-428 and L-1236 were embedded in 0.5% agarose gel. After formalin fixation and paraffin embedding, 2 μm thick sections were cut, allowing an objective assessment of expression intensities in small and giant HRS cells. The same primary antibodies as well as additional antibodies for LDHB (LS-B6870, Lifespan, dilution 1:400) and HSPA8 (GTX111150, GeneTex, Irvine, CA, USA, dilution 1:100) were used with the Vectafluor Excel Amplified Dyelight 594 Anti-rabbit or Anti-mouse kit (Vector laboratories, Burlingame, CA, USA). Respective negative controls showed no specific staining. Immunofluorescence was evaluated on an Axioskop 2 fluorescence microscope with an AxioCam MRm camera (both Zeiss). Expression intensities were then analyzed using ImageJ software (http://imagej.nih.gov).

### Fluorescence activated cell sorting and flow cytometry

The HL cell line L-428 was sorted according to the cell size observed in the forward scatter or with respect to the CD15/CD30 expression pattern using a fluorescence activated cell sorter (FACS) Aria II (Beckton Dickinson, Heidelberg, Germany). Sorted populations were confirmed and further analyzed by a MACSQuant analyzer (Miltenyi Biotech, Bergisch Gladbach, Germany). Staining was performed by standard procedure using antibodies against CD15 coupled with FITC (Miltenyi Biotech, Bergisch Gladbach, Germany) and CD30 coupled with PE (Miltenyi Biotech, Bergisch Gladbach, Germany). Sorted cells were cultured separately for 14 days and the composition of small and big cells as well as CD15/CD30 expression pattern was assessed every 3–4 days. For sorting of different cell sizes, the main population was divided according to cell size into equal numbers of small and big cells (each 20%) with a clear gap (60% of the live cells) to delineate the different populations.

### Colony forming assays

After cell sorting, 600 cells were pipetted into 3.3 ml RPMI 1640 medium containing 1% methylcellulose, 30% FBS, 2% L-glutamine, 1% penicillin/streptomycin and 0.5 μM β-mercaptoethanol. Duplicates of 1.1 ml were platted into 35 mm culture dishes and incubated for 7 days in a standard cell culture incubator to obtain single clones (colonies) of 10–25 cells. Colonies were then counted manually by conventional microscopy.

### Apoptosis assay and Brentuximab Vedotin treatment

After cell sorting, 1 x 10^5^ cells were seeded per ml into 24-well plates with 1 ml of total volume. For assessment of Brentuximab Vedotin induced cell death, the drug was added at total concentrations of 10, 25, or 50 μg/ml and cell numbers as well as the apoptosis rates were assessed using the APC-Annexin V Apoptosis Detection Kit (eBioscience, San Diego, Kalifornien, USA) at 48h.

### Size exclusion of HRS cells

To dissect giant cells from the bulk culture, a volume of 80–100 ml L-428 cell culture was filtered through a cell strainer with 50 μm pore size (Fisher Scientific, PA, USA). The cell strainer was then washed two times with 20 ml PBS reducing the amount of contaminating small cells. Next, the cell strainer was inverted and flushed with medium. Giant cells were collected in a 10 cm culture dish and transferred into 24- or 6-well plate for further culture or directly analyzed.

### Statistical analysis

For analysis of cell numbers and fluorescence intensities, data was tested for the presence of a Gaussian distribution by a Kolmogorov-Smirnov-test. If a Gaussian distribution was present, a two-tailed unpaired t-test was applied, otherwise a Mann-Whitney-test was performed.

## Results

### Cell size and surface marker expression correlate with HRS cell subpopulations

**Fig 1:**
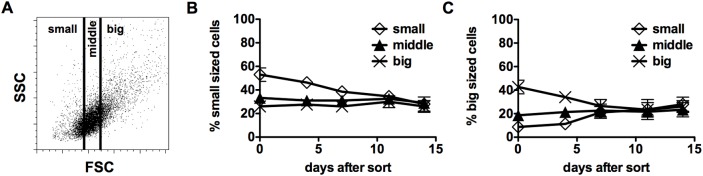
Analysis of L-428 cells sorted by cell size or CD15/CD30 expression. (A) L-428 cells were sorted by cell size (small, middle, big) or after staining for CD15 and CD30 into CD15+CD30+ double positive (DP) and CD15-CD30+ single positive (SP) cells. Frequency of (B) small- and (C) big-sized cells within the three different populations was recorded over time via fluorescence activated cell sorting (FACS).

First, we were interested to determine if small and big HRS cells differ in their immunophenotype. For this purpose, L-428 cells were sorted according to cell size (small, medium and big, Fig 1) and afterwards analyzed for CD15/CD30 expression (S1 Fig). In the subgroup enriched of big L-428 cells the amount of double positive (DP) cells was increased, whereas the number of CD30 single positive (SP) cells was decreased. This finding implicates that larger sized L-428 cells more often express CD15 together with CD30 than their smaller counterpart. In addition, the opposite experiment was performed. L-428 cells were flow sorted into SP and DP cells (S1 Fig) and further analyzed for cell size via FACS (S1 Fig). In line with the previous experiment, SP cells showed the highest amount of small-sized cells and the DP population had an increased frequency of big-sized cells.

Both approaches led to the same result, namely that DP L-428 cells are larger in size than the CD30 single positive counterpart. However, neither by sorting according to cell size nor via marker profile, a pure giant or small cell population could be obtained. Cell size and CD15/CD30 expression were monitored in the enriched populations over time. Reanalysis showed that the frequencies of small-sized or big-sized cells were adjusted within all enriched populations after 5–10 days (Fig 1).

### Big HRS cells show a significantly reduced clonal growth potential

**Fig 2:**
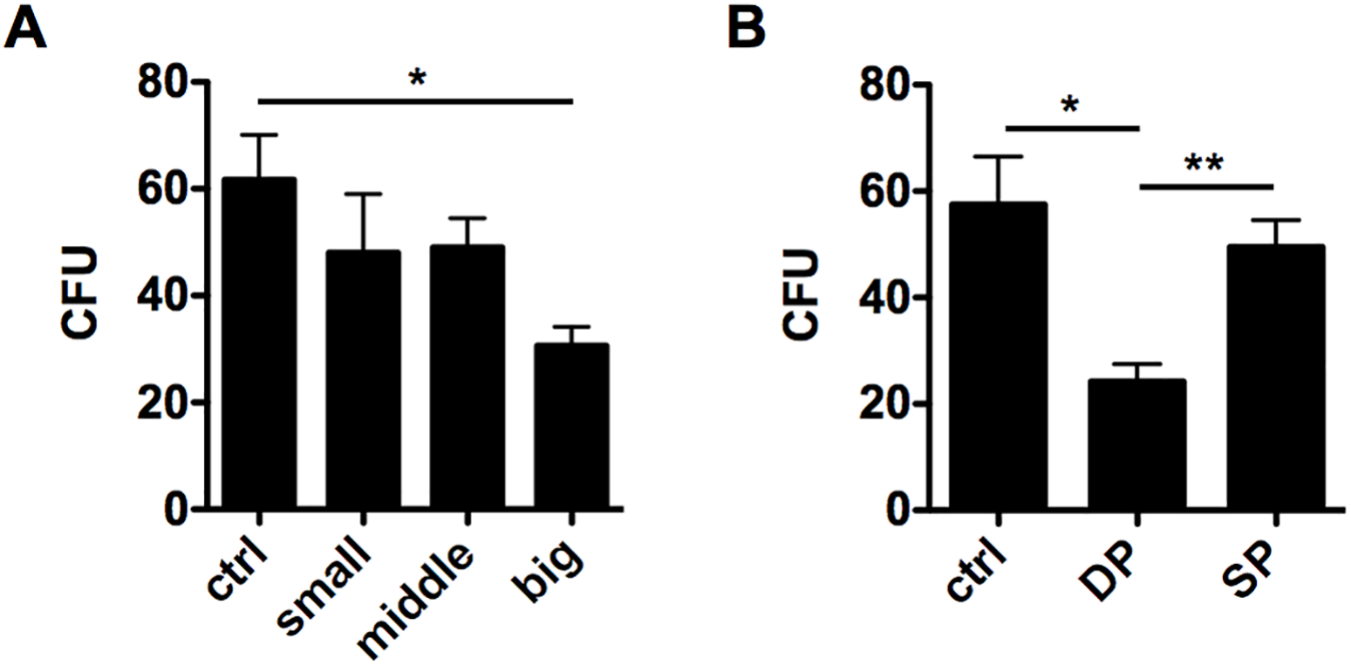
Big DP L-428 cells have a reduced clonal growth potential. L-428 cells were sorted by (A) cell size (small, middle, big) or (B) CD15/CD30 expression (SP, DP), and subsequently used for a colony formation assay (CFA). After one week, colony forming units (CFU) were counted compared to the bulk population. CFA were performed in duplicates and repeated three times.

As a next step, a colony formation assay (CFA) was chosen to elucidate clonal growth potential of individual cells of the sorted subpopulations. In fact, a significantly reduced clonal growth potential could be detected for big-sized as well as DP L-428 cells (Fig 2).

The fast reconstitution of HRS cell subpopulations sorted via cell size might be due to the higher fragility of giant HRS cells and sorting of these cells could increase cell death within the big-sized population. Hence, another more gentle strategy to separate L-428 cells by cell size was applied. Cells were filtered through a 50 μm-cell strainer retaining giant cells (> 50 μm in diameter). Interestingly, size-exclusion led to a significantly increased frequency of DP cells in the big-sized population, again confirming that big L-428 cells are more frequently DP (S2 Fig). Conventional microscopy of the isolated giant cell population confirmed the usually big size of the isolated cells. However, small cells were frequently attached to the giant cells (data not shown). These heterogeneous clusters may be responsible for the results at reanalysis after 7 days, when the amount of DP cells within the big-sized population was markedly reduced (S2 Fig), especially as the proliferation capacity of small HRS cells remains more potent. Although size exclusion experiments revealed the most pure HRS-cell subpopulation with regard to cell size, purity was not sufficient enough for further analysis, i.e. gene expression profiling. Hence, a more precise isolation method was chosen for further experiments.

### Small and big HRS cells lack major differences in gene expression

**Fig 3:**
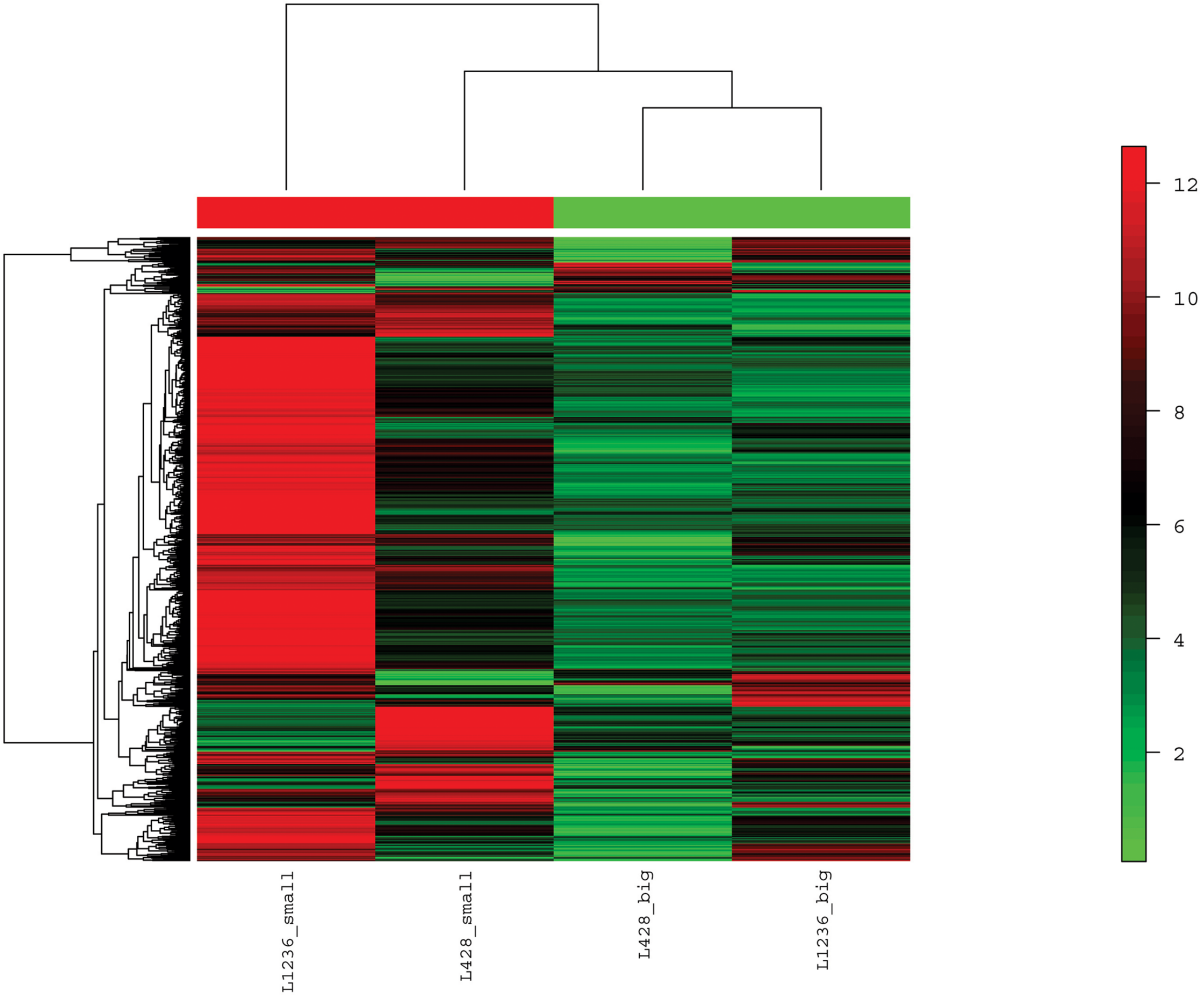
Unsupervised hierarchical clustering of gene expression profiles obtained from microdissected small and giant HRS cells of the cHL cell lines L-428 and L-1236 shows a strong heterogeneity between the two cell lines. Small HRS cells of both cell lines show more strongly expressed probesets (red color) than giant HRS cells. 958 differentially expressed probesets between the four samples representing a standard deviation of > 1 were considered for the analysis.

Small and giant HRS cells were microdissected from smears of the cHL cell lines L-428 and L-1236. Isolated cells were then processed for microarray analysis. Unsupervised hierarchical clustering of gene expression arrays revealed differences between the four samples investigated. In general, among the differentially expressed probesets between the four samples, small HRS cells showed a higher number of over expressed probesets (mean 672) than giant HRS cells (mean 162, Fig 3). In a supervised comparison of small HRS cells to giant HRS cells from both cell lines, only two significantly differentially expressed genes were found when a stringent filtering with a false discovery rate (FDR) of 0.1 was applied. These were split hand/foot malformation (ectrodactyly) type 1 (*SHFM1*, 34-fold upregulated, p<0.01) and lactate dehydrogenase B (*LDHB*, 27-fold upregulated, p<0.01). When less stringent filter criteria (p<0.05 and FDR<0.3) were applied, thirteen genes were found to be upregulated in small compared to giant HRS cells, including six ribosomal proteins and one eukaryotic translation initiation factor (Table 1), suggesting that small HRS cells are more active in protein synthesis. There were no genes that were significantly overexpressed in the giant HRS cells when compared to small HRS cells.

**Table 1:** Genes differentially expressed between small and big HRS cells of Hodgkin cell lines L-428 and L-1236.

Fold change	p-value	False discovery rate	Gene Symbol	Gene Description	GO Biological Process Term
53.5	0.035	0.292	RPS17	ribosomal protein S17	rRNA processing // translation
37.3	0.024	0.265	RPL24	ribosomal protein L24	ribosomal large subunit assembly // translation // exit from mitosis /
**34.3**	**0.001**	**0.054**	**SHFM1**	**split hand/foot malformation (ectrodactyly) type 1**	**proteolysis**
27.5	0.030	0.276	RPL23A	ribosomal protein L23a	translation
**26.6**	**0.001**	**0.054**	**LDHB**	**lactate dehydrogenase B**	**lactate metabolic process // pyruvate metabolic process // glycolysis**
21.4	0.012	0.232	EIF4B	eukaryotic translation initiation factor 4B	translation
17.5	0.027	0.265	HSPA8	heat shock 70kDa protein 8	protein folding // post-Golgi vesicle-mediated transport // cellular membrane organization
12.7	0.014	0.232	RPL39	ribosomal protein L39	translation
11.8	0.021	0.265	PTMA	prothymosin, alpha	transcription
11.4	0.022	0.265	RPL36AL	ribosomal protein L36a-like	translation
11.4	0.010	0.232	YWHAE	tyrosine 3-monooxygenase/tryptophan 5-monooxygenase activation protein, epsilon polypeptide	protein targeting // apoptosis
9.5	0.038	0.292	HNRNPA1	heterogeneous nuclear ribonucleoprotein A1	nuclear mRNA splicing, via spliceosome // mRNA processing
7.3	0.008	0.232	RPL23A	ribosomal protein L23a	translation

Filter criteria of a p-value <0.05 and a false discovery rate <0.3 were applied. In **fat**, the two genes are displayed, which showed a false discovery rate <0.1.

### SHFM1, LDHB, and HSPA8 proteins are expressed in small and big HRS cells in primary cHL cases as well as in cHL cell lines

**Fig 4:**
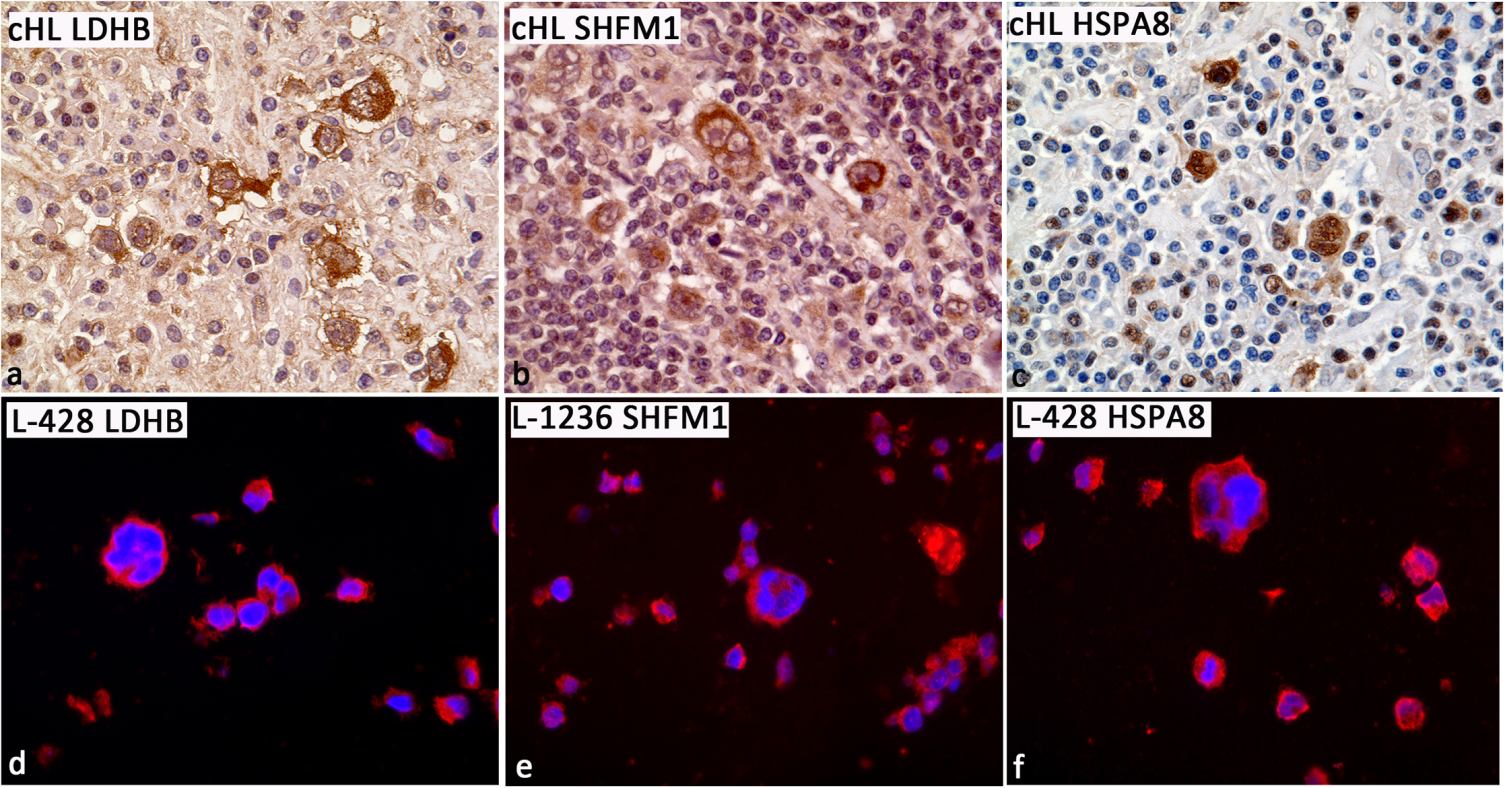
LDHB, SHFM1, and HSPA8 proteins are expressed in small and giant HRS cells. a. LDHB protein expression in small and big HRS cells in a representative primary cHL case (LDHB-immunostaining, 400x). b. SHFM1 protein expression in small and big HRS cells in a representative primary cHL case (SHFM1-immunostaining, 400x). c. HSPA8 protein expression in small and big HRS cells in a representative primary cHL case (HSPA8-immunostaining, 400x). d. Representative example of LDHB expression in small and big HRS cells in the cHL cell line L-428 (LDHB-immunofluorescence staining, 400x). e. Representative example of SHFM1 expression in small and big HRS cells in the cHL cell line L-1236 (SHFM1-immunofluorescence staining, 400x). f. Representative example of HSPA8 expression in small and big HRS cells in the cHL cell line L-428 (HSPA8-immunofluorescence staining, 400x).

Among the differentially expressed genes (Table 1), *SHFM1*, *LDHB*, and *HSPA8* were selected. Primary cHL cases were immunostained with antibodies against these three proteins and a strong expression was observed in the HRS cells in all cases tested (10/10, Fig 4). To be able to better quantify the expression intensities of SHFM1, LDHB, and HSPA8 proteins, the cHL cell lines L-428 and L-1236 were FFPE embedded and immunofluorescence stainings for SHFM1, LDHB, and HSPA8 proteins were performed (Fig 4). The expression intensities were quantified. However, there were no significant differences in the mean signal intensity per area between small and big HRS cells for SHFM1 (S3 Fig), and the expression differences between small Hodgkin and big RS cells observed for LDHB and HSPA8 applying one antibody, could not be confirmed when a different antibody was used (S3 Fig). This indicates that the differences detected by microarray on transcription level did not convincingly translate into changes at the protein level.

### Small and big HRS cells do not show significant differences in susceptibility towards Brentuximab Vedotin treatment

**Fig 5:**
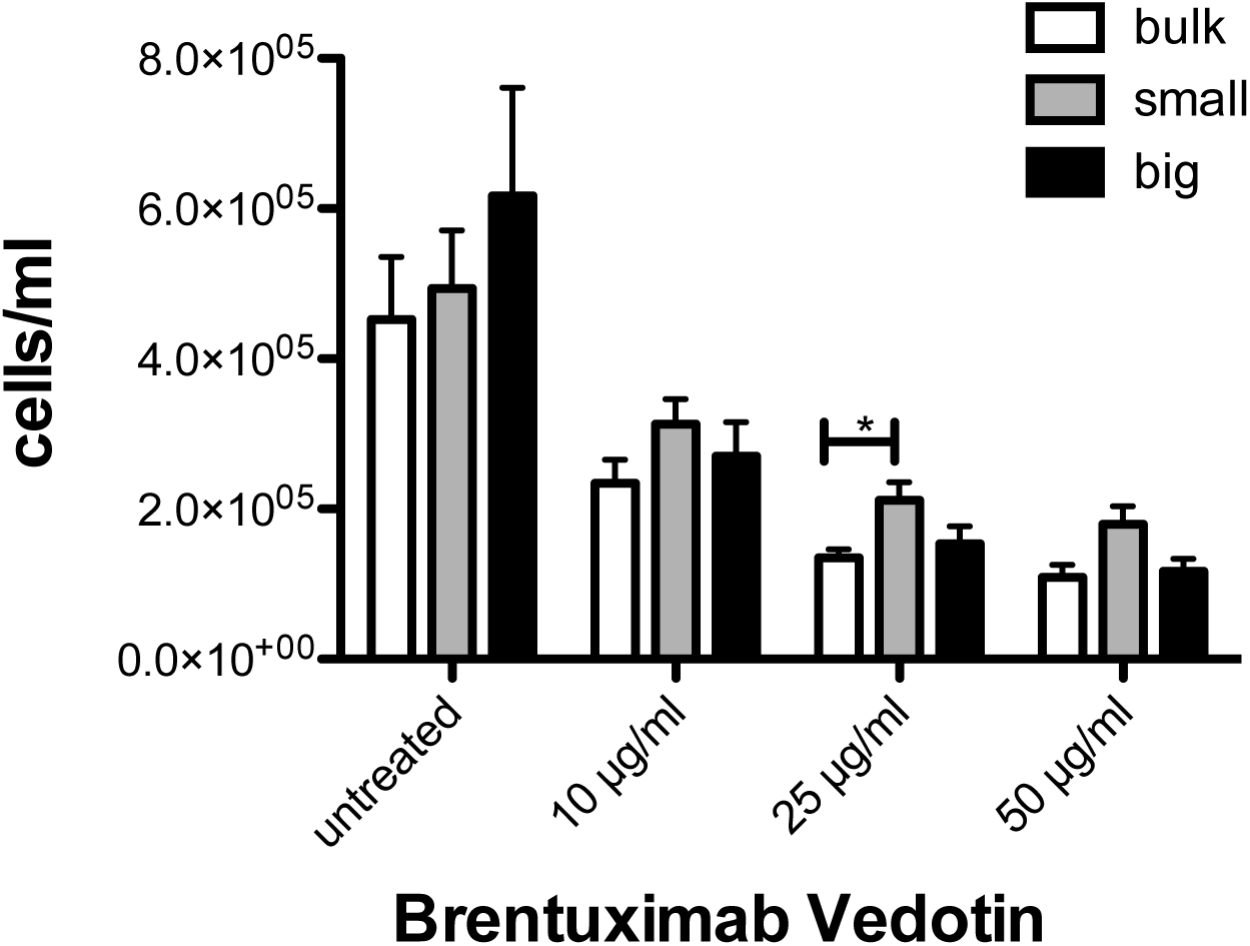
Effect of the anti-CD30 drug conjugate Brentuximab Vedotin to L-428 cells. Cells from the cHL line L-428 were sorted according to FSC in subpopulations of small and big cells (each 20% of total live cell population). Total live gates were sorted as control (bulk). Thereafter, subpopulations were seeded at a density of 1 x 10^5^ cells per ml in triplicates and the indicated amount of Brentuximab Vedotin was added. After 48 h cell numbers were determined by FACS. * p = 0.0117, unpaired t-test.

While first experiments could demonstrate differences between small and big HRS cells, GEP analyses in combination with immunohistochemistry-based validation failed to clearly discriminate between both cell types, although cells were separated according to size with the best available method with respect to purity. Therefore, we also assessed their differential drug response. For this purpose, cell sorting of the cHL cell line L-428 was performed, as a high amount of viable HRS cells separated into small and big subpopulations was needed. Fourty-eight hours after treatment with Brentuximab Vedotin, a trend towards higher cell numbers in the subset of small HRS cells of the L-428 could be detected. However, this difference was only significant at a concentration of 25 μg/ml when small HRS cells were compared with the bulk population (Fig 5, unpaired t-test, p = 0.0117) and was never significant when small HRS cells were compared with big HRS cells. There were no significant differences in the apoptosis rate (data not shown). Taken together, small HRS cells are not significantly more susceptible to Brentuximab Vedotin treatment when compared with big HRS cells.

## Discussion

Giant HRS cells are of high importance in HL, because it is suggested that they shape the tumor microenvironment to support disease progression [14–17]. However, these cells display a differentiated end-stage showing a highly impaired ability to proliferate [10, 18–20], which could be confirmed by colony formation assays in the present study. As a consequence, precursor cells, that are commonly neglected during diagnostic analysis of biopsy specimens, must be responsible for the maintenance of the tumor cell clone [21]. It would be desirable to identify such a stem cell-like HRS cell population, as eradication of these cells would display a way to cure patients suffering from HL [22, 23].

Different groups using different isolation strategies described the presence of HL stem cells within HL cell lines, but unfortunately further validation of the disease-promoting properties of these cells is still missing [8, 9, 24]. Moreover, only Jones *et al*. were able to detect a type of HL stem cells, namely CD20+ HRS cells, also circulating in the blood of HL patients [24]. Although these cells were first identified in HL cell lines L-428 and KM-H2, isolation and functional characterization of this subpopulation was not performed to date. We tested different MACS and FACS strategies to isolate CD20+ L-428 cells and an enrichment of CD20+ HRS cells could be obtained (data not shown). Unfortunately and in contrast to the published data, the sorted population had no enhanced clonal growth or proliferation potential (data not shown). Apart from a stem cell-like subpopulation of HRS cells, it would also be desirable to isolate a highly pure subpopulation of giant HRS cells in order to understand which genes drive these cells into incomplete cytokinesis. Although it has been shown that overexpression of the Epstein-Barr virus (EBV)-encoded latent membrane protein 1 induces formation of multinucleated HRS cells [25, 26], EBV-negative cHL cases likewise present bi- and multinucleated giant tumor cells. In the present study we assessed differences in gene expression between the small and giant HRS cells of the two cHL cell lines L-428 and L-1236. However, apart from the heterogeneity between the two cell lines, only very little differences were observed between small and big HRS cells, indicating that there is no unifying feature between the small and the big HRS cells of these two cell lines.

The most interesting differentially expressed gene found was *SHFM1*, which interacts with *BRCA2* [27]. *BRCA2* has been published to impair the completion of cell division by cytokinesis and to be involved in the formation of aneuploid giant cells [28]. However, in an according study these results could not be reproduced [29]. In contrast, *SHFM1* knock out variants of the yeast *S*. *pombe* also showed a defect in completion of cell division [27]. Downregulation of *SHFM1* in giant HRS cells could therefore be related with the giant size and the bi- or multinucleated phenotype of these cells. However, we could not demonstrate any consistent differences in SHFM1 protein levels between small and big HRS cells and therefore the variable size of these cells is probably not influenced by SHFM1 protein expression. In a recent study differences of the localization of Cyclin A in mononucleated Hodgkin and multinucleated RS cells could be observed [30]. Differences in protein localization may of course also contribute to giant cell formation and were not assessed in the RNA expression profiling in the present study.

When the distribution of small and giant HRS cell populations in cell culture was monitored over time, it was noted that after several days a status quo in the fraction of small and big HRS cells was achieved, indicating that generation of big HRS cells follows a constant mechanism and is a phenomenon intrinsic to the entire HRS cell population. Therefore, the lack of major differences in the gene expression of these two cell populations is consistent with the observed capacity to reconstitute the respective other cell type under culture conditions. In line with this, there were no significant differences between small and big HRS cells in the susceptibility towards Brentuximab Vedotin treatment.

## Conclusions

In summary, we show that small and giant HRS cells have a strong similarity: They lack major differences in gene expression. The generation of big HRS cells follows a constant mechanism as sorting experiments showed a stable reconstitution of the respective subpopulations, explaining the high similarity found in gene expression analysis. Furthermore, susceptibility towards Brentuximab Vedotin treatment is not significantly different when small HRS cells are compared with big HRS cells. The only biological difference observed between small and big HRS cells is a reduced clonal growth potential of big compared to small HRS cells by colony forming assays.

## Supporting information

S1 Fig Big L-428 cells show the highest amount of DP cells. (A) L-428 cells were sorted by cell size (small, middle, big) or after staining for CD15 and CD30 into CD15+CD30+ DP and CD15-CD30+ SP cells. (B+C) Compared to the bulk population, populations sorted for cell size were analyzed for CD15 and CD30 expression by FACS. (B) Frequency of CD15-CD30+ SP cells. (C) Frequency of CD15+CD30+ DP cells. (D+E) Compared to the bulk population, DP and SP cells were analyzed for cell size by FACS. Frequency of (E) small-, (F) middle- and (G) big-sized cells. Experiments were repeated three times.
(PDF)

S2 Fig DP L-428 cells separated by size-exclusion. L-428 cells were placed onto a 50 μm cell strainer. Cells smaller than 50 μm (small) passed the filter, whereas cells bigger than 50 μm (big) were retained. Subsequently, the separated populations were analyzed for CD15/CD30 expression. (A) Exemplary dot blots of small and big L-428 cells. Frequency of DP (B) and SP cells (C) within the two different populations, compared to the bulk population. (D-E) Frequencies were reassessed after 7 days in culture. Experiments were repeated three times.
(PDF)

S3 Fig Quantification of immunofluorescence stainings for LDHB, SHFM1 and HSPA8 in small and big HRS cells of the cell lines L-428 and L-1236. Whereas a significant difference in mean fluorescence intensity between small Hodgkin and big RS cells of the L-1236 was observed with one antibody against HSPA8 (B, GeneTex antibody, p<0.05, t-test), this was not confirmed when a different antibody was applied (A). A significant difference in mean fluorescence intensity between small Hodgkin and big RS cells was also found in the L-428 cell line with an antibody against LDHB (D, antibody LS-B6870, p<0.001, t-test). However, it was not confirmed when a different antibody against LDHB was used (C, antibody LS-B4366). No differences in mean fluorescence intensity were observed for SHFM1 (E).
(JPG)
