# Supplementary figures and images for: Small and big Hodgkin-Reed-Sternberg cells of Hodgkin lymphoma cell lines L-428 and L-1236 lack consistent differences in gene expression profiles and are capable to reconstitute each other
Source: PLoS One. 2017 May 15;12(5):e0177378. doi: 10.1371/journal.pone.0177378 (PMC5432067; doi:10.1371/journal.pone.0177378)

**A**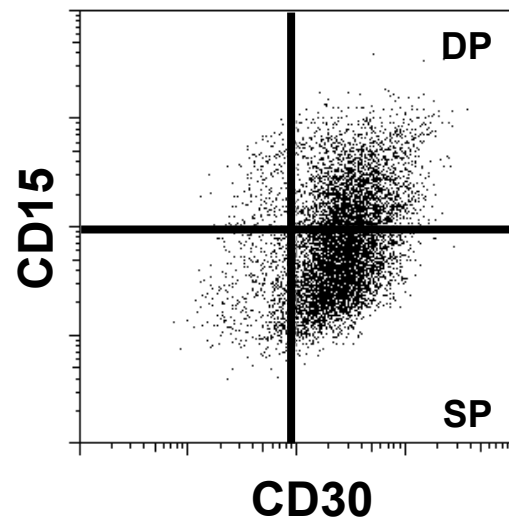**B**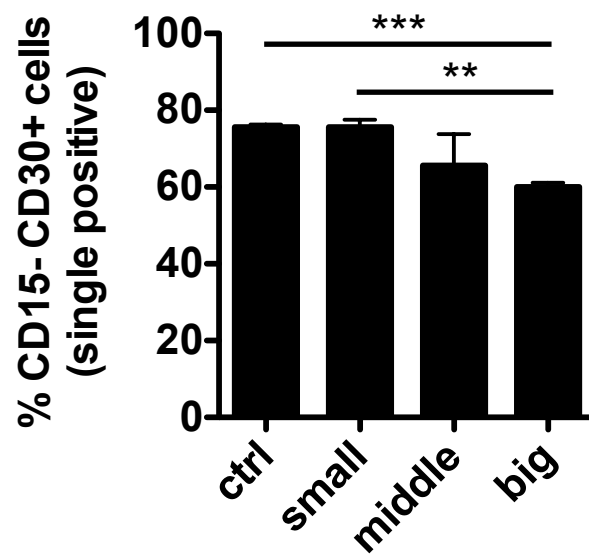**C**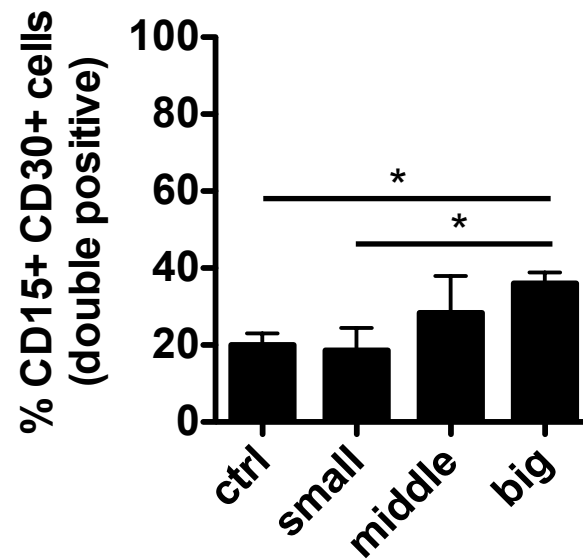**D**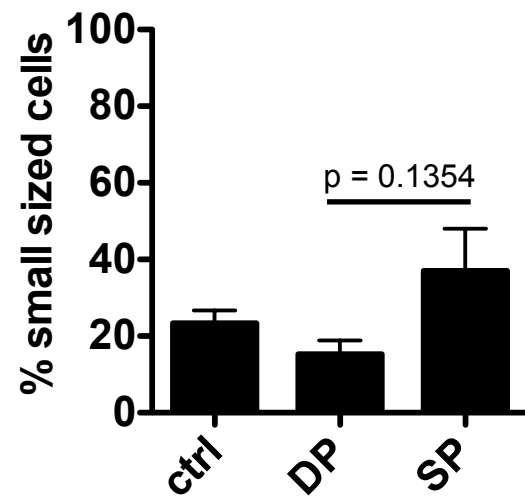**E**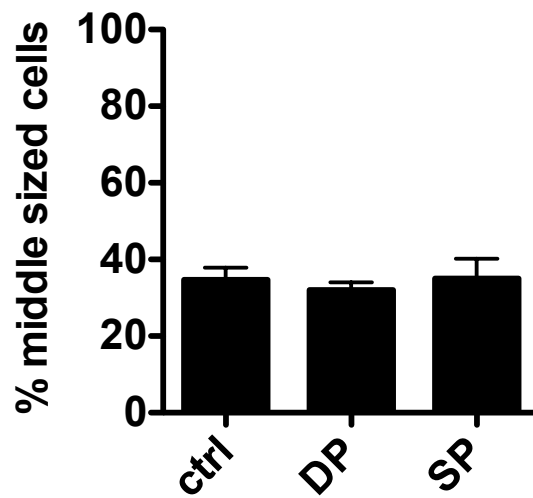**F**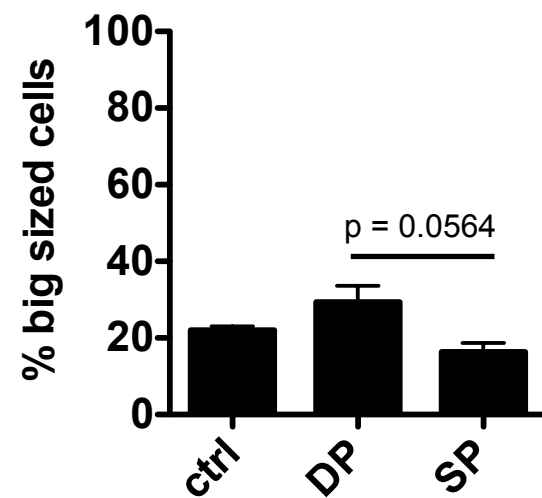

Supplement: S1 Fig — (A) L-428 cells were sorted by cell size (small, middle, big) or after staining for CD15 and CD30 into CD15+CD30+ DP and CD15-CD30+ SP cells. (B+C) Compared to the bulk population, populations sorted for cell size were analyzed for CD15 and CD30 expression by FACS. (B) Frequency of CD15-CD30+ SP cells. (C) Frequency of CD15+CD30+ DP cells. (D+E) Compared to the bulk population, DP and SP cells were analyzed for cell size by FACS. Frequency of (E) small-, (F) middle- and (G) big-sized cells. Experiments were repeated three times. (PDF) [file pone.0177378.s001.pdf]

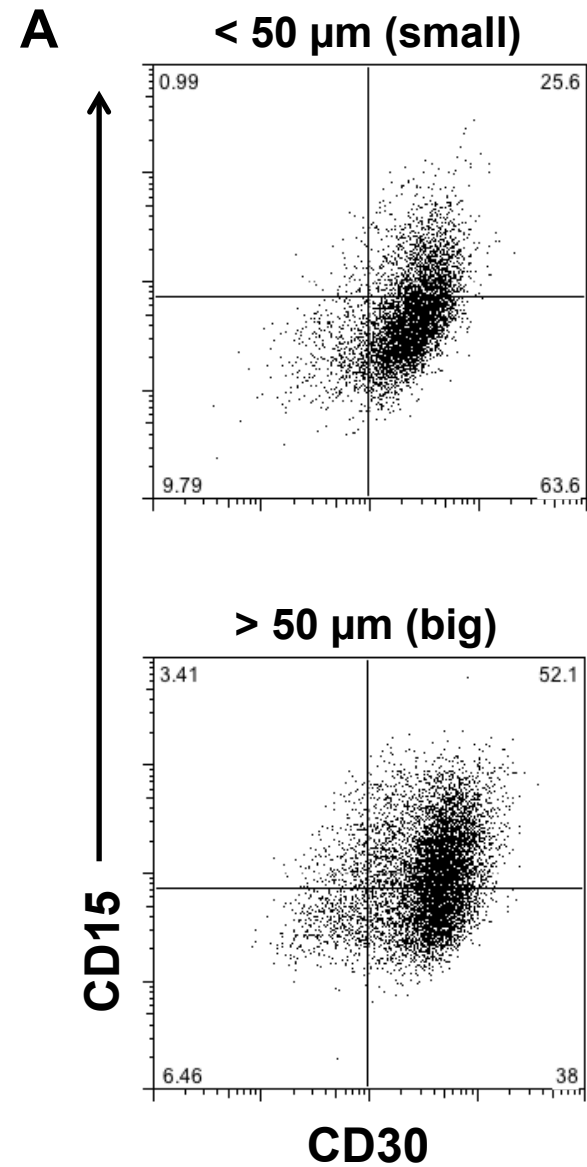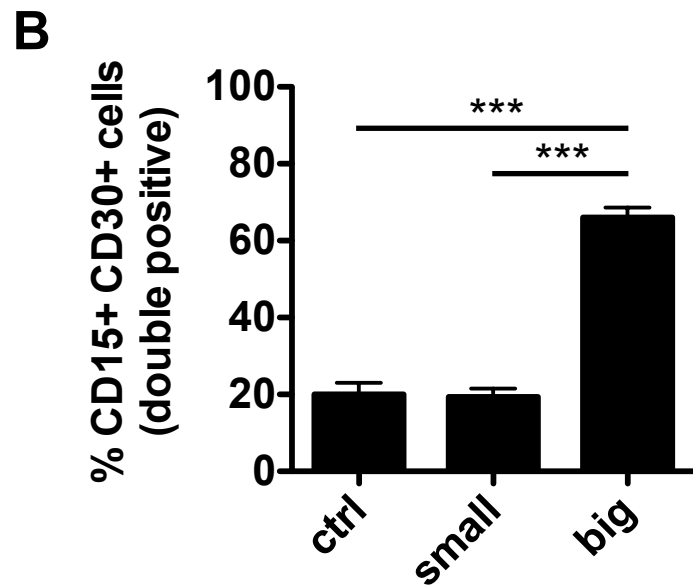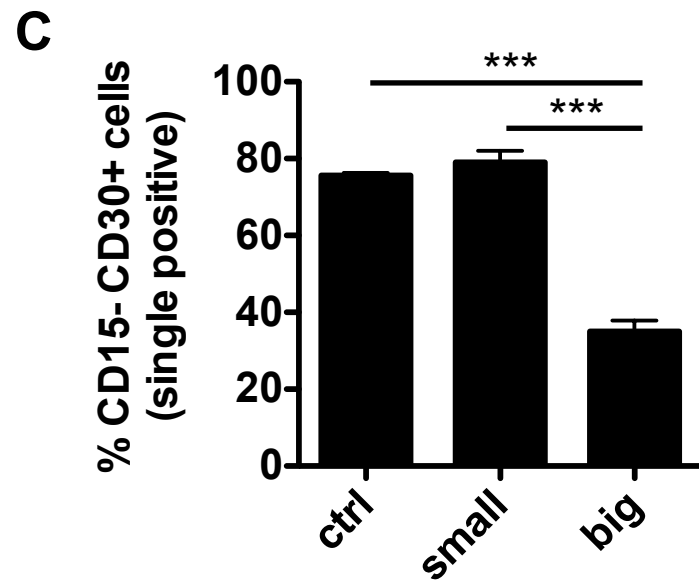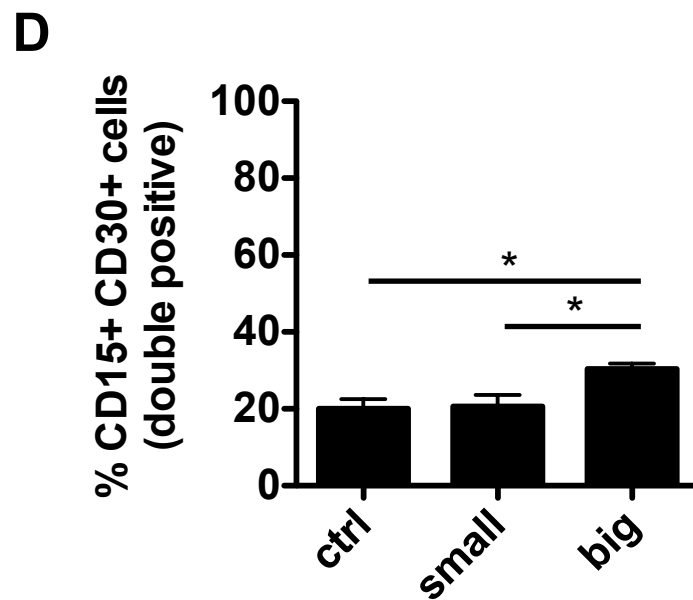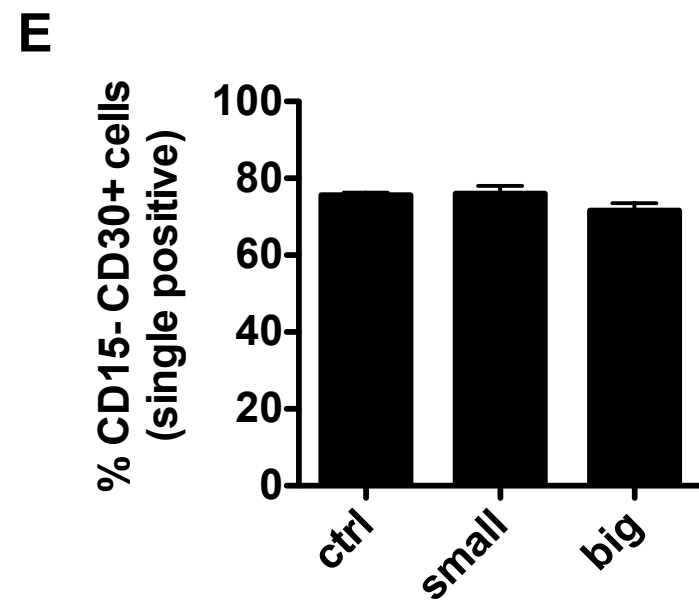

Supplement: S2 Fig — L-428 cells were placed onto a 50 μm cell strainer. Cells smaller than 50 μm (small) passed the filter, whereas cells bigger than 50 μm (big) were retained. Subsequently, the separated populations were analyzed for CD15/CD30 expression. (A) Exemplary dot blots of small and big L-428 cells. Frequency of DP (B) and SP cells (C) within the two different populations, compared to the bulk population. (D-E) Frequencies were reassessed after 7 days in culture. Experiments were repeated three times. (PDF) [file pone.0177378.s002.pdf]

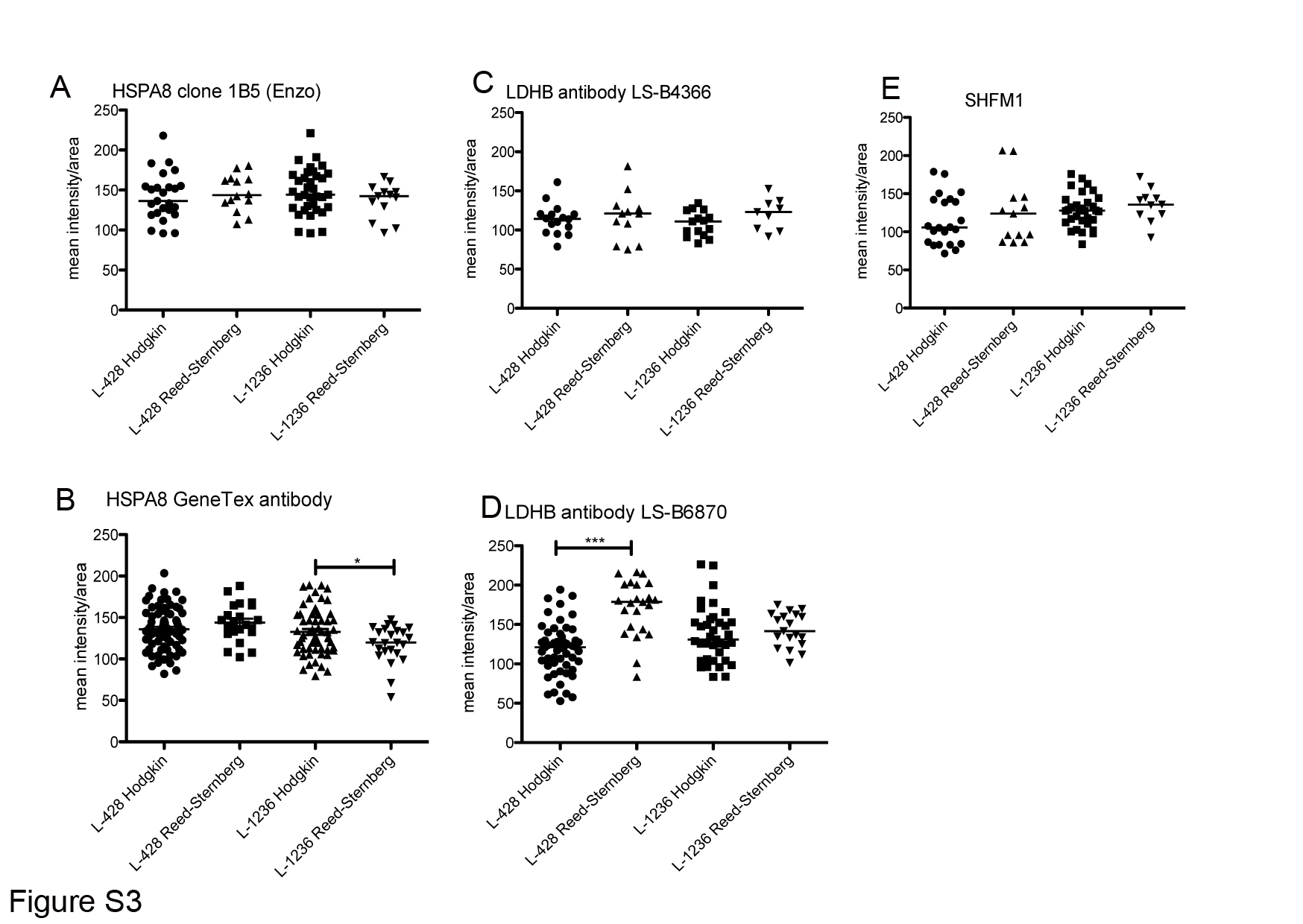

Supplement: S3 Fig — Whereas a significant difference in mean fluorescence intensity between small Hodgkin and big RS cells of the L-1236 was observed with one antibody against HSPA8 (B, GeneTex antibody, p<0.05, t-test), this was not confirmed when a different antibody was applied (A). A significant difference in mean fluorescence intensity between small Hodgkin and big RS cells was also found in the L-428 cell line with an antibody against LDHB (D, antibody LS-B6870, p<0.001, t-test). However, it was not confirmed when a different antibody against LDHB was used (C, antibody LS-B4366). No differences in mean fluorescence intensity were observed for SHFM1 (E). (JPG) [file pone.0177378.s003.jpg]
